# Association between presbycusis and cognitive function: contributing factors in older adult

**DOI:** 10.3389/fnmol.2026.1820873

**Published:** 2026-09-15

**Authors:** Duo Wei, Yuehong Chen, Bin Liu, Zhiqiang Tan

**Affiliations:** Department of Otolaryngology Head and Neck Surgery, Hunan Provincial People’s Hospital and The First Affiliated Hospital of Hunan Normal University, Changsha, China

**Keywords:** cognitive dysfunction, hearing loss, Mini-Mental State Examination, presbycusis, speech recognition

## Abstract

**Background and Objectives:**

This cross-sectional study investigated the association between presbycusis (PC) and cognitive function in older adults and identified factors influencing cognitive performance.

**Materials and Methods:**

A total of 126 participants ( ≥ 60 years) were enrolled, including 58 with PC and 68 controls with normal hearing. Cognitive function was assessed using the Mini-Mental State Examination (MMSE). Statistical analyses included *t*-tests, chi-square tests, Spearman’s correlation, and binary logistic regression.

**Results:**

MMSE scores declined significantly with age in both groups (PC: *r* = −0.585; control: *r* = −0.572, both *p* < 0.001). In the PC group, lower MMSE scores correlated with higher pure-tone average (*r* = −0.563, *p* < 0.01) and poorer speech recognition (*r* = 0.365, *p* = 0.01). Logistic regression identified higher education as an independent protective factor against cognitive impairment (OR = 0.162, 95% CI: 0.031–0.842, *p* = 0.021), whereas greater hearing loss severity (OR = 1.083, 95% CI: 1.001–1.172, *p* = 0.046) and longer hearing loss duration (OR = 0.778, 95% CI: 0.646–0.937, *p* = 0.008) were associated with increased odds of cognitive impairment. Age and sex were not significant.

**Conclusions:**

Greater hearing loss severity, poorer speech recognition, and longer hearing loss duration are associated with lower cognitive function, while higher education is protective. These findings support the close interrelationship between auditory and cognitive health in older adults, suggesting that early audiological assessment and hearing rehabilitation may aid cognitive health, though causality requires longitudinal validation.

## 1 Introduction

Against the backdrop of a globally aging population, rapid socioeconomic and medical advancements, and increased life expectancy, presbycusis (PC) has garnered significant attention (Slade et al., 2020). PC, or age-related hearing loss, is a leading cause of chronic disability and ranks among the most prevalent conditions in older adults (Lin et al., 2011). Characterized by progressive bilateral high-frequency hearing loss and declining speech discrimination, it represents the most common sensory disorder in this demographic (Lin et al., 2011). In China, older adults with PC constitute approximately 34.1% of all individuals with auditory disorders (World Health Organization, 2021). The functional decline of sensory and neural elements leads to slowed central auditory processing, especially in noisy environments (Humes et al., 2012), and consequently impairs communication (Rönnberg et al., 2013). These challenges significantly diminish quality of life and are associated with social isolation, increased fall risk, and reduced life expectancy (Kamil et al., 2016).

Accumulating evidence suggests that PC is not merely a peripheral sensory deficit but also an independent risk factor for cognitive decline and dementia. However, the precise neurobiological mechanisms linking PC to cognitive impairment remain incompletely understood. Several explanatory hypotheses have been proposed. First, the sensory deprivation hypothesis posits that chronic auditory input loss leads to structural and functional reorganization of brain regions traditionally devoted to auditory processing, potentially at the expense of cognitive networks, thereby accelerating cognitive decline. Second, the cognitive load (or effortful listening) hypothesis suggests that individuals with PC must allocate disproportionate attentional and executive resources to decipher degraded auditory signals, leaving fewer cognitive reserves for higher-order processes such as memory and comprehension. Third, the information degradation hypothesis emphasizes that impoverished or distorted auditory input results in lower-quality semantic representations, which directly impairs encoding and retrieval in verbal memory tasks independent of general cognitive capacity (Wayne and Johnsrude, 2015). Fourth, the cognitive reserve hypothesis proposes that individual differences in lifelong cognitive enrichment (e.g., education, occupational complexity, social engagement) may modulate the expression of PC-related cognitive deficits, such that higher reserve buffers against the effects of hearing loss (Stern, 2012).

Given the high prevalence of PC among Chinese older adults and the potential modifiability of hearing loss through interventions such as hearing aids or cochlear implants, clarifying the relationship between PC and cognitive impairment has important clinical and public health implications. This study aimed to analyze the relationship between presbycusis and cognitive impairment in a representative sample of Chinese older adults and to investigate the factors—particularly those reflecting the above mechanistic pathways—that influence this association. We hypothesized that PC would be significantly and independently associated with cognitive impairment, and that this association would be moderated by indicators of cognitive reserve, and mediated by measures of effortful listening and social isolation.

## 2 Materials and methods

### 2.1 Study participants

This cross-sectional study recruited participants from the Otolaryngology Department of the People’s Hospital of Hunan between January 2021 and December 2022. Given the cross-sectional design, this study can only identify associations, not causal relationships.

We enrolled 126 adults aged ≥ 60 years, including 58 patients diagnosed with presbycusis (PC) and 68 controls with normal hearing. Inclusion criteria for the PC group were: (1) age ≥ 60 years; (2) diagnosis of presbycusis according to the “Expert Consensus on Diagnosis and Intervention of Hearing Loss in the Elderly (2019)”; (3) mentally competent and self-sufficient; (4) fluent in Mandarin; (5) no history of dizziness, otitis media, or prior ear surgery. Inclusion criteria for the control group were identical except for having normal hearing. Exclusion criteria for both groups included: history of neurological or psychiatric disorders, use of ototoxic medications, and uncorrected visual impairment that would interfere with cognitive testing. A convenience sampling method was used, and no formal sample size calculation was performed.

Data collected included sex, age, education level, duration of hearing loss, and hearing aid use. Participant characteristics are summarized in Table 1.

**TABLE 1:** Basic characteristics of the two groups.

Factor	PC group (*n* = 58)	Control group (*n* = 68)
Sex		
Male	27 (46.6)	36 (62.1)
Female	31 (53.4)	32 (37.9)
Age (years)		
60–69	30 (51.7)	52 (76.4)
70–79	22 (38.0)	11 (16.1)
80–89	6 (10.3)	5 (7.4)
Education level		
Primary school	19 (32.8)	30 (43.8)
Junior high school	23 (39.7)	22 (33.0)
High school and above	16 (27.5)	16 (23.2)
Deafness duration (years)		
0.1–5	20 (35.5)	
5.1–10	29 (50)	
> 10	9 (15.5)	
PTA (hearing loss severity)		
No	58	68 (100.0)
Mild	0	
Moderate	31 (53.4)	
Moderate to severe	11(19.0)	
Severe	16 (27.6)	
Speech discrimination score		
Low	17 (29.3)	
High	41 (70.7)	

Data are presented as *n* (%). PC, presbycusis; PTA, pure-tone average.

### 2.2 Auditory assessment

Pure-tone audiometry was conducted in a soundproof booth using a semi-automated device (Madsen Astera, Denmark). Air conduction thresholds (dB HL) were measured. The pure-tone average (PTA) was calculated as the mean threshold at 0.5, 1, 2, and 4 kHz for each ear, with data from the better-hearing ear (lower PTA) used for analysis. Hearing loss was categorized per the 2021 WHO classification: mild (20–34 dB HL), moderate (35–49 dB HL), moderately severe (50–64 dB HL), severe (65–79 dB HL), profound (80–94 dB HL), and complete deafness ( ≥ 95 dB HL).

Speech discrimination was assessed in quiet using a Mandarin single-syllable word list. The better-hearing ear was tested at 60 dB HL to determine the speech recognition rate and maximum speech recognition rate. A score ≥ 50% indicated useful hearing.

### 2.3 Cognitive function assessment

Cognitive function was screened using the Mini-Mental State Examination (MMSE), a validated screening tool with high specificity and sensitivity for detecting cognitive impairment in older adults. It is important to note that the MMSE is a screening instrument, not a diagnostic tool for dementia or mild cognitive impairment, and it does not differentiate among specific cognitive domains such as episodic memory, executive function, or processing speed. The MMSE assesses orientation (10 points), memory (3 points), attention/calculation (5 points), recall (3 points), and language (9 points), for a total of 30 points. Based on established cut-offs validated in the Chinese elderly population, and accounting for educational level, a score of 27–30 was classified as normal cognitive function, and a score < 27 indicated cognitive impairment. Considering the potential influence of education, subgroup analyses were conducted accordingly.

### 2.4 Statistical analysis

Inter-group differences in continuous variables were analyzed using independent-samples *t*-tests or Mann-Whitney U tests, as appropriate. Categorical variables were compared using chi-square tests. One-way ANOVA was used for comparisons among multiple subgroups. Spearman’s correlation analysis examined relationships between MMSE scores and age, PTA, speech recognition rate, and disease duration. Binary logistic regression analysis was performed to evaluate the association of sex, age, hearing loss duration, hearing loss severity, and education level with cognitive function (dichotomous outcome: normal/impaired). Results were reported as odds ratios (OR) with 95% confidence intervals (CI). A two-tailed *p* value < 0.05 was considered statistically significant. All analyses were performed using SPSS version 26.0.

## 3 Results

### 3.1 Participant characteristics and general findings

The PTA thresholds for all PC participants ranged from 36.25 to 75 dB HL. MMSE scores exhibited a significant negative correlation with age in both the control group (*r* = −0.572, *p* < 0.001) and the PC group (*r* = −0.585, *p* < 0.001). Comparative MMSE scores across age subgroups are detailed in Table 2.

**TABLE 2:** MMSE scores by age group for control and PC groups.

Age (years)	MMSE scores	
	Control group	PC group
60–69	29.39 ± 1.18	28.68 ± 1.16
70–79	29.18 ± 0.72	27.65 ± 1.25
80–89	27.40 ± 0.41	26.17 ± 1.25
*p*	< 0.001	< 0.001

Data are presented as mean ± standard deviation. MMSE, Mini-Mental State Examination; PC, presbycusis. Differences in MMSE scores between the PC and control groups were statistically significant across all three age cohorts, as illustrated in Figures 1A–C.

**FIGURE 1:**
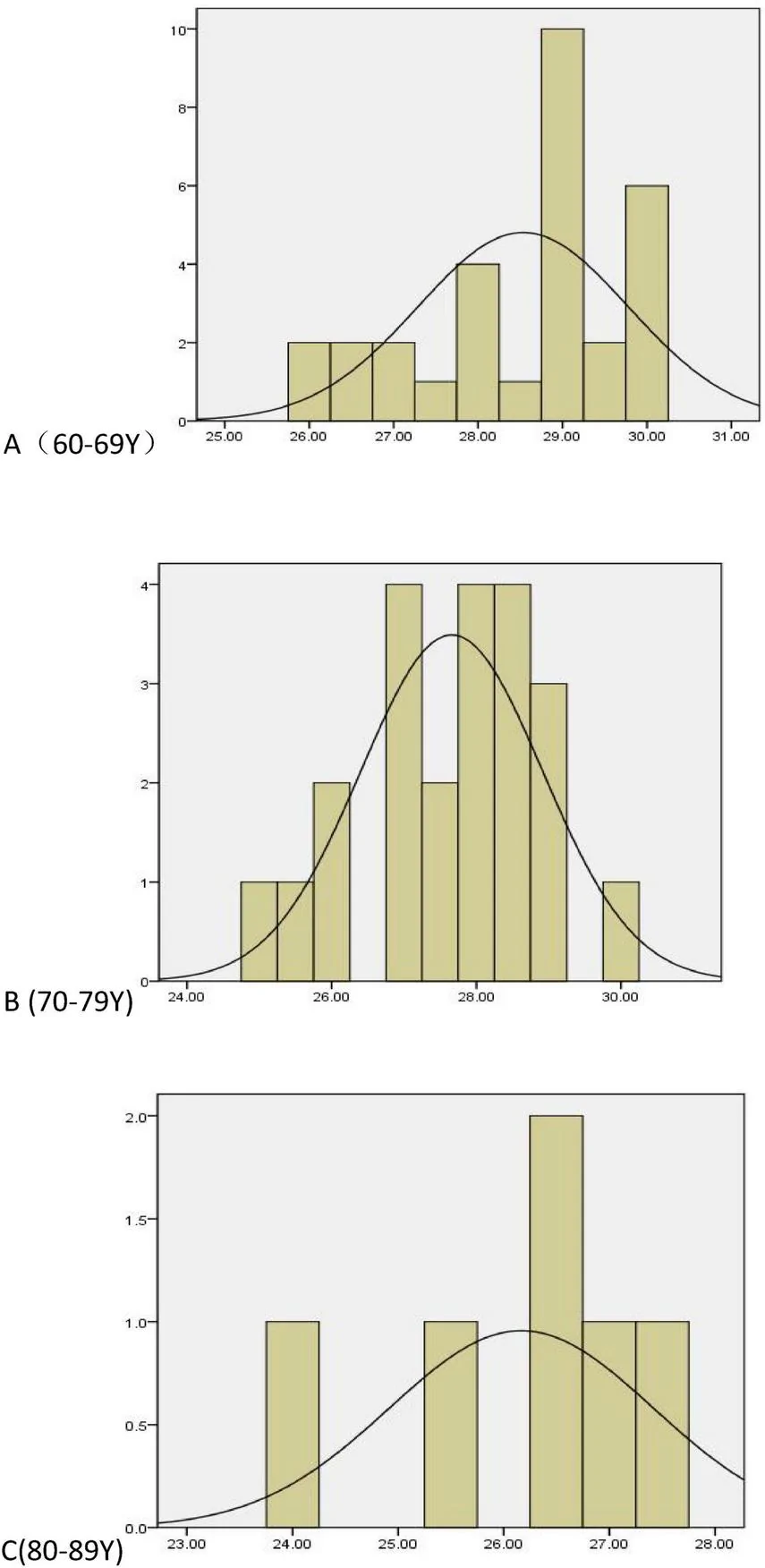
Groups were statistically significant across all three age cohorts, as illustrated in **A–C**.

### 3.2 Correlations between MMSE Scores, hearing thresholds, and speech recognition

In the PC group, a significant negative correlation was found between MMSE scores and PTA threshold (*r* = −0.563, *p* < 0.01; Table 3). A significant positive correlation was observed between MMSE scores and maximum speech recognition rate (*r* = 0.365, *p* = 0.01; Table 4).

**Figure:**
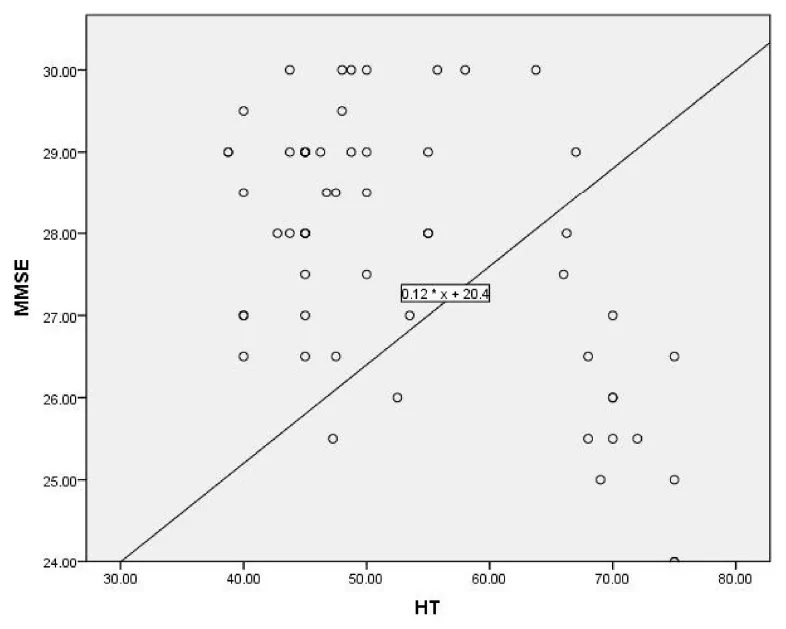


**TABLE 3:** Comparison of MMSE scores across different average hearing threshold levels.

Group	*n*	MMSE score (points)
Control group	68	29.21 ± 1.00
PC group		
PC group(Mild to moderate-Severe)	45	28.40 ± 1.19
PC group (Severe)	23	27.38 ± 1.95

Data are expressed as mean ± standard deviation.

**TABLE 4:** Comparison of MMSE scores across different speech recognition rates.

Speech recognition rate (%)	*n*	MMSE score (Points)
Low (< 50%)	17	26.12 ± 1.45
High (≥ 50%)	41	28.48 ± 1.10

Data are expressed as mean ± standard deviation.

### 3.3 Factors associated with cognitive impairment in PC

Binary logistic regression analysis (Table 5) identified a higher education level (OR = 0.162, 95% CI: 0.031–0.842, *p* = 0.021) as an independent protective factor against cognitive impairment. Greater hearing loss severity (OR = 1.083, 95% CI: 1.001–1.172, *p* = 0.046) and longer hearing loss duration (OR = 0.778, 95% CI: 0.646–0.937, *p* = 0.008) were significantly associated with higher odds of cognitive impairment. Age (OR = 0.896, 95% CI: 0.749–1.071, *p* = 0.227) and sex (OR = 1.155, 95% CI: 0.929–1.436, *p* = 0.182) were not statistically significant factors in this model.

**TABLE 5:** Logistic regression analysis of factors associated with cognitive impairment in the PC group.

Variable	β	OR	95% CI	*p-value
Age	-0.110	0.896	0.749–1.071	0.227
Sex (male vs. female)	0.144	1.155	0.929–1.436	0.182
Education level (higher vs. lower)	-1.820	0.162	0.031–0.842	0.021*
Hearing loss severity (per dB)	0.080	1.083	1.001–1.172	0.046*
Hearing loss duration (per year)	-0.251	0.778	0.646–0.937	0.008*

β, regression coefficient; OR, odds ratio; CI, confidence interval.

*p < 0.05.

## 4 Discussion

Our findings contribute to the growing body of evidence linking presbycusis to cognitive decline in older adults. The present study identified significant associations between hearing loss severity, hearing loss duration, speech recognition ability, and cognitive function as measured by the MMSE, while also confirming the protective role of higher educational attainment. These results warrant careful interpretation within the framework of existing explanatory hypotheses and prior empirical findings.

The progressive decline in MMSE scores observed across advancing age groups in both the PC and control cohorts (Table 2) is consistent with the well-established trajectory of age-related cognitive changes (Harada et al., 2013). However, the consistently and significantly lower MMSE scores in the PC group compared to age-matched controls–evident even in the youngest cohort (60–69 years: 28.68 ± 1.16 vs. 29.39 ± 1.18)–suggest that hearing loss may compound the effects of normal aging on cognition. From a clinical perspective, this finding implies that cognitive screening should be routinely integrated into the audiological assessment of older adults, even those in their seventh decade of life, rather than being deferred until more advanced ages.

Several complementary, non-mutually exclusive hypotheses have been proposed to explain the association between age-related hearing loss and cognitive decline. Our findings provide differential support for these frameworks, which warrants critical examination. The sensory deprivation hypothesis posits that prolonged reduction in auditory input leads to structural and functional brain reorganization, including accelerated cortical atrophy in temporal and frontal regions, with cascading effects on cognitive processes (Lin et al., 2014; Peelle et al., 2011). Our observation that longer hearing loss duration was associated with greater cognitive impairment (OR = 0.778 per year, 95% CI: 0.646–0.937, *p* = 0.008) is consistent with this hypothesis, as prolonged deprivation would be expected to result in more pronounced neuroplastic changes. Notably, 50% of our PC participants had a hearing loss duration of 5.1–10 years, and 15.5% exceeded 10 years, suggesting that many individuals are living with unaddressed hearing loss for extended periods. Each additional year of hearing loss was associated with approximately 22% higher odds of cognitive impairment in our model. This temporal gradient, while derived from cross-sectional data and therefore not proof of causation, reinforces the public health imperative of reducing the time between symptom onset and intervention. Current delays in help-seeking for hearing loss—often reported to average 7–10 years in the literature (Davis et al., 2007)–may represent a modifiable window of opportunity for cognitive preservation. However, the sensory deprivation hypothesis has been challenged by longitudinal studies showing that the cognitive trajectory after cochlear implantation or hearing aid fitting does not always normalize to age-expected levels, suggesting that auditory deprivation alone may not fully account for the observed cognitive deficits (Amieva et al., 2015).

The increased cognitive load hypothesis proposes that the continuous effort required for perceptual processing in degraded auditory conditions depletes finite cognitive resources, leaving fewer reserves available for higher-order functions such as working memory and executive control (Tun et al., 2009). Our finding that speech recognition ability—a measure sensitive to the perceptual effort required for auditory decoding—correlated with MMSE scores (*r* = 0.365, *p* = 0.01) offers indirect support for this model. Patients with low speech recognition scores ( < 50%) exhibited markedly lower MMSE scores (26.12 ± 1.45) compared to those with high recognition scores (28.48 ± 1.10). This nearly 2.4-point difference on the MMSE, while modest in absolute terms, may represent a clinically meaningful shift in functional status for older adults (Andrews et al., 2019). However, it should be noted that the MMSE is a global cognitive screen and lacks the sensitivity to isolate the specific executive and attentional domains that the cognitive load hypothesis predicts would be most affected (Tombaugh and McIntyre, 1992). Studies employing dual-task paradigms and neuropsychological batteries targeting executive function would be better positioned to test this hypothesis directly.

The information degradation hypothesis emphasizes the downstream consequences of chronically impoverished auditory input on semantic memory and linguistic processing (Wayne and Johnsrude, 2015). While our speech recognition data are broadly consistent with this perspective, our cross-sectional design cannot determine whether degraded auditory input precedes cognitive decline, whether incipient cognitive impairment impairs speech perception, or whether both processes interact reciprocally over time. This bidirectional possibility is increasingly recognized in the literature, with Lin and colleagues proposing a ‘vicious cycle’ model in which hearing loss and cognitive decline mutually exacerbate one another (Lin et al., 2013).

The cognitive reserve hypothesis posits that individual differences in cognitive processing efficiency–shaped by factors such as education, occupational complexity, and lifelong cognitive engagement–provide resilience against the clinical expression of neuropathology (Stern, 2002, 2012). Our identification of higher education level as an independent protective factor (OR = 0.162, 95% CI: 0.031–0.842, *p* = 0.021) directly supports this hypothesis. Importantly, education remained significantly associated with better cognitive outcomes after controlling for hearing loss severity and duration, suggesting that cognitive reserve may operate independently of, rather than merely through, auditory pathway integrity. This finding resonates with Stern’s foundational work on cognitive reserve (Stern, 2002) and with the Lancet Commission’s identification of education as a key modifiable factor in dementia risk reduction (Livingston et al., 2020). However, educational attainment is a static proxy for cognitive reserve; our estimated protective effect (OR = 0.162) should be interpreted cautiously given our modest sample size and the resulting wide confidence interval. Future studies should incorporate dynamic measures of cognitive and social engagement throughout the lifespan (Valenzuela and Sachdev, 2006).

Our results align with prior research in several important respects. Davies et al. reported that the incidence of cognitive impairment was 1.6 and 1.4 times higher in individuals with severe and moderate hearing loss, respectively, compared to those with normal hearing (Davies et al., 2017). Similarly, we observed significantly lower MMSE scores in the PC group across all age strata, consistent with Huh’s report of cognitive decrements in older adults with hearing impairment (Huh, 2018). However, direct comparison of effect sizes across studies is complicated by differences in cognitive assessment tools, hearing loss classification systems, and analytic approaches. Notably, our use of the 2021 WHO hearing loss classification (World Health Organization, 2021) represents a more contemporary grading system than that employed in many earlier studies, which may affect the comparability of severity-stratified results.

PTA reflects peripheral auditory sensitivity, while speech recognition engages higher-order auditory and cognitive processing (Humes et al., 2012). Ren et al. suggested that speech recognition may be more closely tied to cognition than pure-tone thresholds (Ren et al., 2024). Our observed positive correlation between MMSE scores and maximum speech recognition rate supports this view, as effective speech comprehension demands cognitive resources for signal extraction and organization (Pichora-Fuller et al., 2016). The dynamic interplay between auditory and cognitive systems in supporting speech-in-noise perception has been well documented (Anderson et al., 2013a). The modest correlation coefficient (*r* = 0.365), however, indicates that speech recognition explains only a limited portion of the variance in MMSE scores, and other factors–both auditory and non-auditory–undoubtedly contribute to cognitive outcomes. From a clinical perspective, speech recognition testing may serve a dual purpose in audiological practice: assessing communicative function and identifying patients who may benefit from formal cognitive evaluation. Furthermore, the observation that speech recognition–a measure integrating peripheral and central auditory processing–correlates with cognition underscores the importance of rehabilitation strategies that extend beyond simple amplification to include auditory training programs designed to optimize speech-in-noise perception and central auditory processing (Anderson et al., 2013b). Declining speech recognition can lead to communication breakdown, social withdrawal, reduced cognitive stimulation, and potentially accelerated decline, highlighting the critical need for early PC diagnosis, timely auditory rehabilitation, and cognitive training (Mick et al., 2014).

We confirmed that greater hearing loss severity is associated with lower MMSE scores, consistent with Li’s findings in a Chinese elderly population (Zhang et al., 2022). A longer hearing loss duration was also significantly associated with cognitive impairment, suggesting that prolonged auditory deprivation may compound risk over time, a pattern consistent with the sensory deprivation hypothesis (Lin et al., 2014). This finding underscores the importance of early intervention. Management should extend beyond hearing aids to include psychological support and facilitated social engagement to enhance quality of life and cognitive vitality (Maharani et al., 2019).

Education level emerged as a significant protective factor, supporting the cognitive reserve hypothesis (Stern, 2012). Higher education is often linked to greater cognitive resilience, possibly due to more mentally stimulating lifestyles and greater neurobiological reserve capacity (Wilson et al., 2013). Encouraging older adults with PC to engage in lifelong learning, social activities, and cognitive games may help bolster cognitive reserve and emotional well-being. Fostering supportive social environments represents a recommended public health strategy to mitigate cognitive decline in this vulnerable population (Livingston et al., 2020).

## 5 Conclusion

In this cross-sectional study of older adults with presbycusis, greater hearing loss severity and longer hearing loss duration were significantly associated with lower cognitive function as measured by the MMSE, whereas higher education level was independently associated with better cognitive performance. These findings, while not establishing causation, suggest that auditory and cognitive health are closely interrelated in older adults. Early audiological assessment, timely provision of hearing rehabilitation, and promotion of cognitively stimulating activities throughout the lifespan may represent complementary strategies for supporting cognitive health in the aging population. Longitudinal research with comprehensive covariate control is needed to determine whether interventions targeting hearing loss can modify cognitive trajectories.

## 6 Limitations

This study has several important limitations that must be carefully considered when interpreting the findings. First, the cross-sectional design precludes any causal inferences. The temporal relationship between hearing loss and cognitive impairment cannot be determined from our data. It is equally plausible that incipient cognitive decline impairs auditory processing, that hearing loss contributes to cognitive deterioration, or that a bidirectional relationship exists (Lin et al., 2013). Longitudinal studies with repeated measures of both auditory and cognitive function are essential to disentangle the directionality of these associations. Second, potential selection bias exists due to single-center recruitment from an otolaryngology department. Our sample likely over-represents treatment-seeking individuals and may not generalize to community-dwelling older adults with unaddressed hearing loss. Additionally, the healthy volunteer effect may apply to our control group, which was younger on average and demonstrated high MMSE scores (mean 29.21 ± 1.00), potentially inflating the apparent difference between groups (Ganguli et al., 1998). Third, the absence of comprehensive confounding control is a critical limitation. Several variables that may confound the relationship between hearing loss and cognition—including socioeconomic status, physical activity, dietary habits, cardiovascular comorbidities, diabetes, depression, social isolation, and hearing aid use—were not systematically assessed or statistically adjusted for Livingston et al. (2020); Loughrey et al. (2018). The observed associations may therefore partially reflect the influence of these unmeasured factors rather than a direct effect of hearing loss *per se*. The relationship between hearing loss and cognition is embedded within a complex network of health, lifestyle, and social variables that future studies should measure and model comprehensively. Fourth, cognitive function was evaluated using only the MMSE. While this instrument is widely used and validated for cognitive screening, it has well-documented limitations: it is not a diagnostic tool for dementia or mild cognitive impairment; it has ceiling effects that reduce sensitivity to subtle changes in high-functioning individuals; it is influenced by age, education, and cultural background; and it provides a single global score that cannot differentiate among specific cognitive domains such as episodic memory, executive function, and processing speed–domains that may be differentially affected by hearing loss (Mitchell, 2009; Tombaugh and McIntyre, 1992). The use of a more comprehensive neuropsychological battery would enable more nuanced investigation of which cognitive domains are most closely associated with auditory function. Fifth, our relatively small sample size (*n* = 126, including 58 PC patients and 68 controls), combined with the absence of *a priori* sample size calculation, limits statistical power, particularly for subgroup analyses and multivariable modeling. The wide confidence intervals for some estimates (e.g., education level OR: 0.031–0.842) reflect this imprecision and warrant cautious interpretation. Sixth, hearing loss was characterized using behavioral pure-tone audiometry and speech recognition testing. We did not include objective measures of central auditory processing, electrophysiological assessments, or neuroimaging, which could provide additional mechanistic insights (Slade et al., 2020). Similarly, auditory rehabilitation history—including type, duration, and consistency of hearing aid use–was not comprehensively analyzed, despite its potential to modify the hearing loss–cognition relationship (Dawes et al., 2015). Seventh, the lack of ethnic diversity in our sample (all Han Chinese participants from a single geographic region) limits generalizability to other populations.

Despite these limitations, our study provides clinically relevant evidence for the association between presbycusis severity, duration, and cognitive function, and highlights the protective potential of educational attainment. Future research should employ prospective longitudinal designs, multicenter and community-based sampling, comprehensive neuropsychological assessment batteries, and rigorous measurement and statistical control of potential confounders to advance understanding of the mechanistic pathways linking age-related hearing loss to cognitive decline.

## Data Availability

The original contributions presented in this study are included in this article/supplementary material, further inquiries can be directed to the corresponding author.
